# The Treatment of Uterine Displacements

**Published:** 1885-05

**Authors:** 


**The Treatment of Uterine Displacements,** Including Prolapsus, Anteversion, Retroversion, Anteflexion and Retroflexion. By W. EGGERT, M. D. Second edition. Illustrated. Chicago: Duncan Brothers. 1884.

The author presents in this work the homœopathic therapeutics for the uterine diseases and displacements. He says in the preface to the first edition that " all the remedies have been given in the 200th potency and upwards," of which one dose has been given per day." Whether a work is worthy of perusal, which recommends for prolapsus and other displacements, as curative agents, such infinitesimal doses, we leave our readers to learn, if they desire to do so, by sending for a copy.

